# Oscillatory components of bidirectional cardio-respiratory coupling in depression and suicidal ideation: insights from swarm decomposition and entropy analysis

**DOI:** 10.3389/fnetp.2025.1620862

**Published:** 2025-09-23

**Authors:** Herbert F. Jelinek, Mohanad Alkhodari, Ahsan H. Khandoker, Leontios J. Hadjileontiadis

**Affiliations:** ^1^ Department of Medical Sciences, Khalifa University, Abu Dhabi, United Arab Emirates; ^2^ Health Engineering Innovation Group, Khalifa University, Abu Dhabi, United Arab Emirates; ^3^ Cardiovascular Clinical Research Facility, Radcliffe Department of Medicine, University of Oxford, Oxford, United Kingdom; ^4^ Electrical and Computer Engineering-Aristotle University, Thessaloniki, Greece

**Keywords:** network physiology, cardio-respiratory coupling, heart rate variability (HRV), swarm decomposition, fractal dimension, major depressive disorder (MDD)

## Abstract

**Introduction:**

Major depressive disorder (MDD) and MDD with suicidal ideation (MDDSI) present with heterogeneous symptoms, complicating diagnosis and treatment. Precision psychiatry addresses this challenge by applying computational methods and digital biomarkers to objectively distinguish psychiatric states. While psychiatric research has traditionally focused on neural activity, increasing evidence highlights the value of autonomic indices, particularly heart rate variability (HRV), in capturing clinically relevant dysregulation. Cardio-respiratory coupling (CRC), which reflects bidirectional interactions between cardiovascular and respiratory systems, represents a physiologically grounded extension of this approach. Although less frequently applied in psychiatry compared to HRV, CRC offers a sensitive window into autonomic network dynamics and holds promise for differentiating between MDD and MDDSI.

**Methods:**

A total of 74 participants were assigned to Control (n = 35), MDD (n = 21), or MDDSI (n = 18) groups. ECG, PPG, and respiratory signals were recorded at rest and segmented into 2-min intervals. Swarm Decomposition (SwD) was applied to extract four oscillatory components (OC1–OC4) from each signal that go from low to high frequency, respectively. Fractal dimension (Higuchi, Katz) and Shannon entropy quantified coupling complexity. Bidirectional (λbi) and unidirectional (λ) coupling measures and phase angles were computed between respiratory signals and cardiovascular markers: pulse wave amplitude (PWA), pulse transit time (PTT), and pulse rate (PR). Group differences were evaluated using Kruskal–Wallis and *post hoc* tests (p < 0.05).

**Results:**

Bidirectional PR coupling in OC3 showed significant group differences (p < 0.01). Higuchi fractal dimension of PTT in OC3 was reduced in MDDSI compared to MDD and controls (p = 0.018), suggesting diminished complexity. For PWA in OC4, high-frequency power significantly differed between controls and MDDSI (p = 0.004). Directional coupling entropy also distinguished MDD from MDDSI (p = 0.039).

**Conclusion:**

This study reveals that frequency-specific disruptions in bidirectional cardiorespiratory coupling, along with reduced signal complexity and entropy, are characteristic of MDDSI. These features may reflect impaired autonomic adaptability and emotional regulation. Phase-based coupling metrics and SwD show promise as physiological biomarkers for early identification of high-risk depressive states in digital psychiatry.

## Introduction

Physiological data, such as EEG, provides an objective perspective into conditions like major depressive disorder (MDD) and suicidality, and is an important biomarker for psychiatrists to identify mental health disorders (Badrakalimuthu et al., 2011; Lebiecka et al., 2018; Liu et al., 2022). MDD affects more than 180 million people worldwide and is a leading cause of disability, with suicide representing one of its most devastating outcomes (Marx et al., 2023). Discriminating between MDD with and without suicidal tendencies, as well as distinguishing both from healthy states, is therefore critical for timely intervention and prevention strategies. Recent methods in biosignal analysis have significantly improved the identification and understanding of psychiatric diseases by including advanced techniques such as multimodal signal decomposition, directional coupling analysis, and fractal and entropy measures (Salankar et al., 2021; Marzbanrad et al., 2020; Jiang et al., 2024; Bartsch et al., 2012; Platiša et al., 2020). These methods provide a detailed perspective of physiological interactions, which are important for understanding the autonomic dysregulation associated with psychiatric conditions.

Network physiology emphasizes the complex, directional, and multiscale interactions between physiological subsystems, offering a system-level lens to understand how distributed organ networks coordinate to maintain homeostasis (Salankar et al., 2021; Chen B. et al., 2022; Borovkova et al., 2022; Khalaf et al., 2015; Bartsch and Ivanov, 2014; Krohova et al., 2019). A disruption in these dynamic interactions reflects a loss of physiological complexity and adaptability, which can serve as an early marker of systemic dysfunction and disease (Ivanov, 2021; Ivanov and Bartsch, 2014; Bashan et al., 2012). Approaches grounded in complexity science (Morandotti et al., 2025), particularly measures of information flow such as transfer entropy, provide a powerful framework for quantifying these network interactions and detecting early deviations from healthy dynamics. In psychiatry, this perspective has gained importance through the rise of digital psychiatry, which leverages physiological signals to complement symptom-based assessments and provide objective insights into mental states (Torous et al., 2021; Vignapiano et al., 2023; Zhao et al., 2019). Within this framework, cardio-respiratory coupling (CRC) has emerged as a paradigmatic example of network physiology, as it reflects the continuous exchange of information between cardiac and respiratory systems mediated by vagal pathways to the sinoatrial node and its modulation of heart rate dynamics (Zhao et al., 2019; Kontaxis et al., 2021). Transfer entropy and related complexity metrics allow the directional information flow within CRC to be quantified, thereby capturing the degree of coordination and adaptability in autonomic regulation. Reduced vagal modulation, commonly indexed by diminished heart rate variability (HRV) and impaired respiratory sinus arrhythmia (RSA), has been repeatedly associated with MDD (Koch et al., 2019; Kemp et al., 2010) and, in some studies, suicidality (Sheridan et al., 2021; Adolph et al., 2018). These findings support the view that impaired autonomic regulation represents a core feature of psychiatric morbidity, while also contributing to the elevated cardiovascular risk observed in MDD populations.

Synchronization between physical subsystems has been widely investigated in physics (Sobiech et al., 2017), and, while early applications to biology, neuroscience, and psychopathology lagged behind (Rosenblum et al., 2002), this has changed considerably over the past 2 decades. Numerous studies have since demonstrated synchronization of physiological rhythms, including heart rate, respiration (particularly during slow breathing), blood pressure, cerebral vascular flow, sympathetic muscle activity, brain oscillations, and pupil dilation, often using phase-locking approaches and synchrograms to characterize coupling (Folschweiller and Sauer, 2021; Andrews et al., 2025; Melnychuk et al., 2021). Coupling of multimodal systems refers to the interaction of two or more oscillatory processes that influence one another through the exchange of information or energy, thereby shaping each other’s dynamics. Coupling can take different forms, strong or weak, unidirectional or bidirectional, linear or nonlinear (Dick et al., 2014). Synchronization, by contrast, describes the temporal alignment of oscillations across systems, such as phase locking, frequency locking, antiphase relations, or synchronization with a delay. Both coupling and synchronization are central features of complex network physiology, including heart-to-brain interaction (HBI), which has been investigated using several correlation and causality algorithms such as Granger causality (GC), transfer entropy (TE), and controlled time delay stability (Marzbanrad et al., 2020; Valenza et al., 2018a; Faes et al., 2013; Porta et al., 2013; Ivanov et al., 2016).

Coupling of biosignals first became popular with the use of Granger causality to characterize functional circuits associated with cognition and behavior in health and disease (Pichot et al., 2024). GC identifies directed functional interactions by implementing a statistical, predictive notion of causality (Seth et al., 2015). Porta and colleagues discussed GC as part of the autonomic network linking heart rate with respiration (Porta et al., 2013). Temporal causality features have also been extended to frequency-based measures, including cross-spectral and information-theoretic approaches, showing cardiorespiratory information coupling (Faes et al., 2021). Other nonlinear approaches have also been applied to cardiorespiratory coupling. For example, cross-sample entropy and multiscale entropy analyses have revealed statistically significant coupling between respiration and interbeat interval variability (Ahmed and Mandic, 2011). By contrast, one recent study employing higher-order detrended moving-average cross-correlation analysis (DMCA) in a small sample (n = 8) failed to detect significant long-range correlations between breathing patterns and interbeat interval variability (Nakata et al., 2021), underscoring the need for larger studies and methodological triangulation.

Transfer entropy (TE) then extended the time and frequency-based unidirectional coupling and improved on the limitations of Granger causality. TE quantifies the direction and strength of coupling between two signals (Bossomaier et al., 2016). It can be applied to non-stationary and nonlinear systems such as the cardiorespiratory system and quantifies the information transferred from a past signal process (e.g., respiration) to a current target signal (e.g., heart rate), independent of the past information obtained from the target signal (heart rate) (Valenza et al., 2018a; Stokes and Purdon, 2017). In contrast, Granger causality (GC) is inherently limited by its reliance on linear autoregressive models and the assumption of Gaussian-distributed noise, making it less effective in detecting nonlinear interactions and dynamics commonly observed in physiological systems (Chen et al., 2023).

Although TE was developed to address several limitations of GC, including sensitivity to bias or high variance, difficulties in interpretation, and inability to capture nonlinear interactions, such issues in GC arise particularly when the underlying data exhibits strong nonlinear correlations, a scenario in which GC tends to perform poorly (Chen Y. et al., 2022). Moreover, TE is computed using an information-theoretic framework based on Shannon entropy and conditional entropy. To address potential bias due to self-matching, surrogate TE was introduced by shuffling the driver time series and subtracting this value from the original TE estimate, thereby ensuring robustness. Alternative bias-reduction strategies, such as corrected conditional entropy (Porta et al., 2001) or permutation-based TE, have been proposed in the literature, and cross-sample entropy remains a valuable complementary method for avoiding self-matching altogether. TE, by contrast, can account for nonlinear and directional dependencies, but it requires substantially larger datasets, is computationally intensive, and its results may be challenging to interpret (Stokes and Purdon, 2017). Thus, while RSA and HRV measures have proven sensitive to depressive phenotypes, traditional coupling metrics such as GC and TE remain limited by assumptions of stationarity, the need for long recordings, or difficulties in disambiguating directionality in nonlinear systems (Pichot et al., 2024; Vicente et al., 2011). To overcome these challenges, we introduce an extended phase-based bidirectional coupling algorithm based on the Niizeki–Saitoh model, which estimates both the strength and direction of influence with lower computational demands (Niizeki and Saitoh, 2018; Niizeki and Saitoh, 2016).

Cardiovascular and respiratory systems exhibit multiscale oscillatory behavior driven by autonomic nervous system dynamics, including low-frequency oscillations associated with sympathetic activity, baroreflex control, and central neural pacemaking drive from the pons (Pfurtscheller et al., 2017; Pfurtscheller et al., 2020), high-frequency components reflecting parasympathetic modulation during respiration, and intermediate bands indicating dynamic interactions between cortical and subcortical regulatory centers (Wang et al., 2025). Swarm Decomposition (SwD) is a novel approach in non-stationary signal decomposition that utilizes swarm intelligence algorithms inspired by biological swarm behaviors, such as predator-prey dynamics, to extract intrinsic frequency bands from physiological time series. This method adaptively separates signals into constituent oscillatory components (OC) based on amplitude, frequency content, and local structural features, which provides better spectral specificity, reduced mode mixing, and better preservation of signal integrity across time (Apostolidis and Hadjileontiadis, 2017; Baltatzis et al., 2017; Ganiti-Roumeliotou et al., 2023). In psychiatric populations, particularly those with MDD and MDD with suicidal ideation (MDDSI), alterations in autonomic regulation often manifest as blunted or chaotic oscillatory patterns (Lehrer and Eddie, 2013). By isolating the OCs, SwD enables frequency-specific coupling analysis, which can identify small disruptions in physiological regulation that standard time-domain HRV metrics may miss and may provide clinicians with information on the link between specific frequency disruptions to functional domains and enable personalized physiological profiling and targeted interventions such as vagal nerve stimulation or paced breathing therapies.

Fractal time series analysis provides a framework for quantifying the complexity and self-similarity of physiological signals across multiple time scales (Yamamoto and Hughson, 1993; Porcaro et al., 2024). In the context of autonomic regulation, it enables the detection of long-range correlations and dynamic fluctuations that are often obscured in conventional linear analyses (Peng et al., 1992; Stanley et al., 1999; Ivanov et al., 1999; Valenza et al., 2018b). Techniques such as entropy analysis, Higuchi and Katz fractal dimension analysis capture the geometric intricacy and temporal irregularity of signals like electrocardiography (ECG) and photoplethysmogram (PPG), that describe the adaptability or rigidity of the underlying network physiology (Cysarz et al., 2000; Raghavendra and Narayana Dutt, 2009; Higuchi, 1988). In MDD, reduced fractal complexity has been associated with diminished autonomic flexibility and impaired emotional regulation (Zitouni et al., 2022; Mandarano et al., 2022). By applying fractal metrics to oscillatory components derived from the Swarm Decomposition, nonlinear dynamical analysis is integrated with network physiology to investigate changes in cardio-respiratory coupling that distinguish MDD from suicidal ideation (Khandoker et al., 2017a).

**FIGURE 1:**
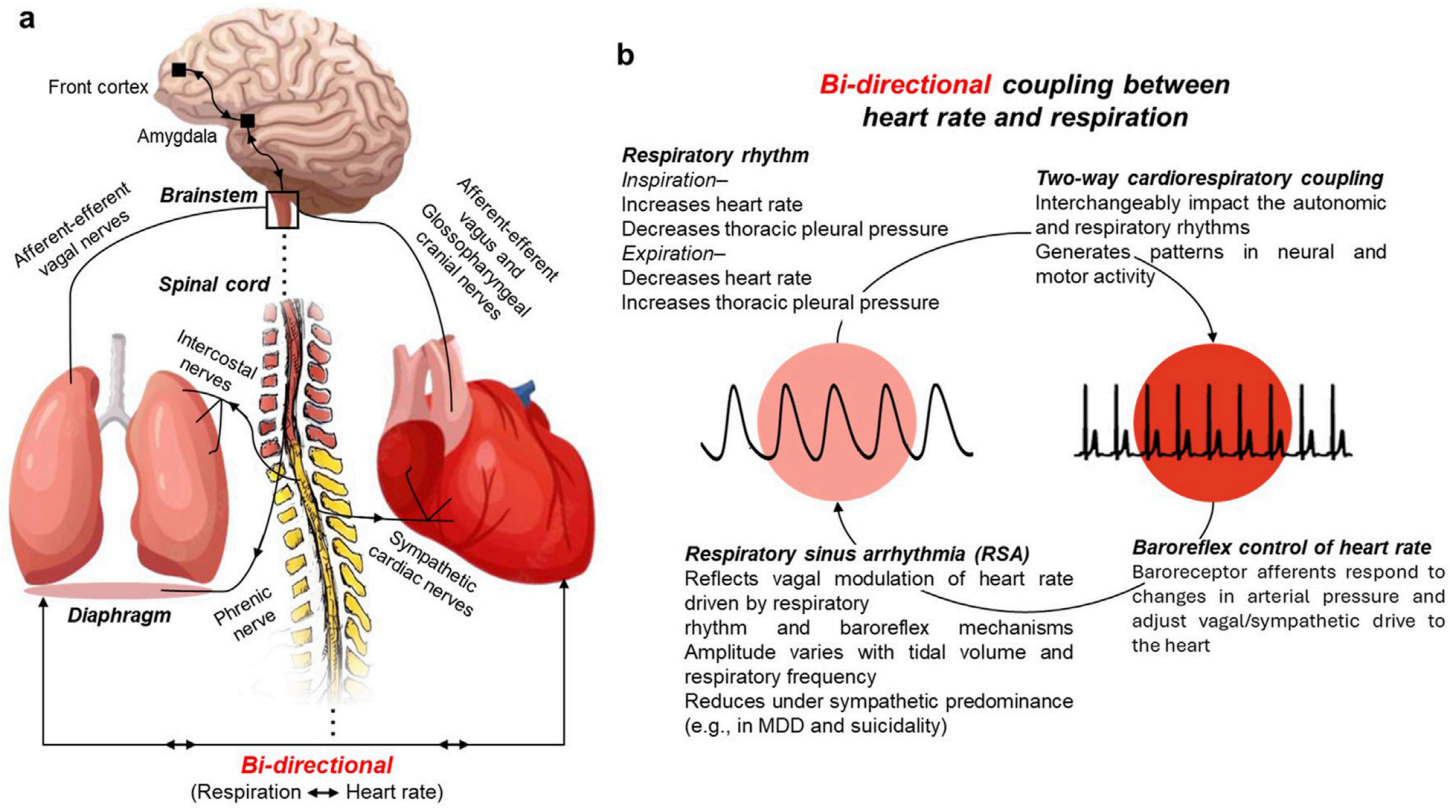
Bi-directional coupling between heart rate and respiration. **(a)** Simplified schematic of neural pathways linking brainstem, heart, and lungs. **(b)** Cardiorespiratory coupling illustrates how respiratory rhythm, baroreflex control, and vagal modulation interact to generate respiratory sinus arrhythmia (RSA). RSA reflects the parasympathetic component of heart rate variability and varies with tidal volume and respiratory frequency. Its contribution to HRV is reduced under sympathetic predominance, as observed in major depressive disorder and suicidality.

Organ networks display bi-directional coherency including between heart rate and respiration (Figure 1). The proposed study extends the phase coherency algorithm discussed by Niizeki and Saito to a bi-directional coupling algorithm that provides phase and directionality to gain a better understanding of the cardio-respiratory coherency in MDD with and without suicidal ideation. To further delineate the biosignal characteristics associated with disease progression, the time series were decomposed into oscillatory components and analyzed using fractal geometry-based methodology.

## Methods

### Demographic and physiological information

Nine demographic variables were recorded for all patients, including age, gender, waist circumference (WC), body mass index (BMI), mean arterial pressure (MAP), Beck Depression Inventory (BDI), general anxiety disorder (GAD-7), and the patient health questionnaire (PHQ-9).

### Dataset and patient enrollment

A total of 74 unmedicated patients were divided into three groups: no major depressive disorder (n = 35; Control), major depressive disorder (n = 21; MDD), and MDD plus suicidal ideation (n = 18; MDDSI). All patients provided informed consent and attended the Abu Dhabi American Centre for Psychiatry and Neurology in the United Arab Emirates (UAE) during the morning hours. The Al Ain District Ethics Committee approved the study. Patient psychiatric history, health questionnaire (PHQ-9), and general anxiety disorder (GAD-7) results were collected. Diagnosis of depression and suicidal ideation was made by the consultant psychiatrist using a structured interview, the mini-international neuropsychiatric interview (M.I.N.I.), and the Hamilton Depression Rating Scale (HAM-D). Patients with significant cognitive impairment, ischemic heart disease, diabetes, psychiatric complications, and any inflammatory illness within the preceding 2 years were excluded from the study. All participants were asked to refrain from drinking coffee and smoking cigarettes before the experiment. However, food intake and physical activities were not restricted to avoid causing anxiety and stress to patients.

### Physiological biosignals acquisition

Physiological signals from each patient were recorded for 10 min in the afternoon, including supine-resting ECG, finger photoplethysmogram (PPG), and respiration. ECG signals were recorded using a lead II configuration (Powerlab, AdInstruments, Australia) with a sampling frequency of 1 kHz. Respiratory and PPG signals were captured using Powerlab and processed on Labchart 7.1 with a sampling frequency of 1 kHz. ECG and PPG signals were filtered with bandpass filters with a frequency range of 0.5–150 Hz and 0.5–15 Hz, respectively.

Only the last 2-min segments from each (10-min) recording were used in this study for further analysis to reduce the occurrence of nonstationarities, ectopic beats, and general noise such as muscle movement. While longer recordings are generally recommended for reliable characterization of nonlinear complexity in physiological signals, shorter segments have practical and methodological value (Shaffer et al., 2020). They are commonly used in clinical practice to assess vital functions and can provide robust discrimination between physiological states, even if they underestimate nonlinear dynamics (Volpes et al., 2022). Moreover, shorter recordings are less demanding for participants and reduce the likelihood of contamination from nonstationarities. In this context, the ability to discriminate between MDD subtypes and healthy controls based on complex analysis of short signals represents an important and practical achievement.

### Decomposing biosignals into oscillatory components

The signals used in this study (ECG, PPG, and respiration) were decomposed into their oscillatory components (OCs) using the recent Swarm decomposition (SwD) algorithm (Alkhodari et al., 2010). SwD is a filtering mechanism with pre-defined parameters that follows a method based on a swarm-prey hunting approach to obtain different components within different frequency ranges. SwD showed a high potential in dividing the ECG and HRV data into multiple components related to the actual known frequency ranges in multiple studies.

In this work, ECG and PPG signals were decomposed into four main OCs that correspond to low, mid-low, mid-high, and high frequency ranges. The filtering parameters were set to 0.03, 0.01, 0.1, and 0.25 for the minimum peak measure, standard deviation of components, Welch window percentage, and clustering factor, respectively. The minimum peak measure affects the total number of components to be extracted, where the lower the value, the higher the number of components. In addition, the Welch window percentage determines how fine or coarse the spectrum will be when included in the algorithm. The clustering factor determines the strength of assigning frequency components into major ones. Finally, each original signal was decomposed into OC1, OC2, OC3, and OC4, which have a range from low to high frequency, respectively. For the respiration signal, the algorithm was used as a denoising method to remove all high-frequency components (OC2, OC3, and OC4) and only select the lowest frequency component (OC1) that represents the respiratory rhythm.

### Extraction of physiological features

Physiological features, including the pulse wave amplitude (PWA), pulse transit time (PTT), and pulse rate (PR) were extracted from ECG and PPG signals. PWA was measured from PPG as sequential values of the amplitudes of peaks in the signal, whereas PTT was calculated as sequential distances between each R-peak and PPG peak in the ECG and PPG signals. PR values were calculated as per the following equation:

$$\mathrm{PR}=\frac{60}{P_{w}}$$

where Pw corresponds to the width of each peak in the PPG signal. It was measured as the distance between peak points (left and right) where the signal intercepts a reference line that equals half of the peak prominence.

Coupling information was extracted between each physiological feature (PWA, PTT, and PR) and the respiratory component, including the angle (degree of coupling), directional coupling (λ), and bi-directional coupling (λ_bi_). The extraction of coupling information was performed for each OC as well as for the original component of each signal.

Coupling information was extracted between pulse rate and the respiratory component including the angle (degree of coupling) and bi-directional coupling (Bi λ). Bi-directional phase coherency was introduced as an extension to the conventional uni-directional phase analysis (Jelinek and Khandoker, 2020) as follows:

$$\lambda \left(t_{k}\right)\left.=\right|\frac{1}{N}\sum_{k-\frac{N}{2}}^{k+\frac{N}{2}}e^{[\phi_{\mathrm{PR}}\left(t_{k}\right)-\phi_{resp}\left(t_{k}\right)]\mathrm{mod}2\pi }{|}^{2}$$

where k denotes the time step in N over all lengths of the selected signals and φ_PR (t_k_) and φ_Resp (t_k_) are the instantaneous phases of the PR and respiratory signals obtained using Hilbert transform. To transform λ(t_k_) into Bi λ(t_k_), we additionally calculated the phase coupling degree of synchronization (Jelinek and Khandoker, 2020) as follows:

$$Bi\;\lambda \left(t_{k}\right)=\lambda \left(tk\right)\times \tan^{-1}\left(\frac{\cos\left(\lambda \left(t_{k}\right)\right)}{\sin\left(\lambda \left(t_{k}\right)\right)}\;\right)$$

Bi λ can be formed ranging from −1 to 1. In this specific scenario, negative coupling indicates (−1 to 0) heart-led interaction, while positive coupling (0–1) indicates respiratory-led interaction (Jelinek and Khandoker, 2020).

A total of 15 features were extracted from the coupling information (Table 1), i.e., the angle (degree of coupling), directional coupling (λ), and bi-directional coupling (λ_bi_) determined from the mean, standard deviation, root mean square of successive differences (RMSSD), Minkowski–Bouligand box-counting fractal dimension (FD), fractal abundance (FA), Higuchi FD, Katz FD, Shannon entropy, high frequency (HF) norm, HF peak, HF power, low frequency (LF) norm, LF peak, LF power, and LF to HF ratio. In addition to the decomposition into four oscillatory components (OC1–OC4), we extracted these standard frequency spectral indices from the coupling information. The goal of this procedure was to enhance discriminatory potential between groups, rather than to interpret each spectral index physiologically in isolation. Power spectral density was estimated using Welch’s method (Shaffer and Ginsberg, 2017; Malik et al., 1996), from which low-frequency (LF: 0.04–0.15 Hz) and high-frequency (HF: 0.15–0.40 Hz) bands were quantified. The LF and HF absolute power, normalized units (LF norm, HF norm), and peak frequencies (LF peak, HF peak) were calculated, along with the LF/HF ratio as a measure of sympathovagal balance.

**TABLE 1:** List of features extracting from the coupling information.

Coupling information	Definition	Clinical translation
Angle (degree of coupling)	Quantifies synchronization strength	Linked to vagal activity and emotional regulation
Directional coupling (λ)	Strength and direction of information transfer	Reduced λ may indicate impaired autonomic control seen in depression
Bi-directional coupling (λbi)	Total bidirectional synchronization	low λbi may reflect breakdown in adaptive communication in depression

Feature	Definition	Clinical Translation
Mean	Average of the coupling values across the recording	Reflects the overall level of synchronization; elevated mean coupling may indicate persistent entrainment, which in pathology could suggest reduced flexibility of regulatory systems
Standard deviation	Measure of dispersion of coupling values around the mean	Captures variability of synchronization; lower SD may reflect reduced adaptability and rigidity in physiological regulation, while higher SD may indicate greater dynamical range of coupling responses
Root mean square of successive differences (RMSSD)	Square root of the mean squared differences between successive coupling values	Short-term variations in Vagal activity
Minkowski–Bouligand box-counting fractal dimension (MBFD)	Estimation of complexity via how coupling data fills space across scales	Lower MBFD indicates rigidity, nonadaptive coupling in pathology
Fractal Abundance (FA)	Quantification of how frequently fractal patterns appear in the coupling signal	Lower FA may suggest loss of dynamical complexity, relevant in depression
Higuchi fractal dimension (HFD)	Estimation of signal complexity	Lower HFD correlates with autonomic dysfunction
Katz fractal dimension (KFD)	Fractal dimension estimate based on ratio of perimeter to maximum distance in the coupling signal	Reduced KFD may reflect diminished adaptability and resilience in physiological control
Shannon entropy	Measure of uncertainty or disorder in coupling strength distribution	Lower entropy signals more predictable, less adaptable systems in pathology
High frequency (HF) norm	Normalized power of high-frequency oscillations	Reduced HF norm indicates parasympathetic withdrawal, often observed in MDD.
High frequency (HF) peak	Dominant peak frequency within the high-frequency band	Shift or reduction in HF peak associated with disrupted vagal modulation
High frequency (HF) power	Total spectral power within the high-frequency band	Reduced HF power reflects lowered vagal input
Low frequency (LF) norm	Normalized power of low-frequency oscillations (mixed sympathetic-parasympathetic influence)	Altered LF norm indicates changes in autonomic balance
Low frequency (LF) peak	Dominant peak frequency within the low-frequency band	Shift in LF peak frequency may signal autonomic dysfunction
Low frequency (LF) power	Total spectral power within the low-frequency band	Changes reflect sympathetic modulation and baroreflex sensitivity
LF to HF ratio	Ratio of low-frequency to high-frequency power	Increased LF/HF ratio indicates sympathetic dominance; decreased ratio suggests parasympathetic dominance

### Statistical analysis of coupling

Prior to group comparisons, data normality was tested, and the majority of features did not follow a normal distribution, thus, a non-parametric approach was applied. Specifically, differences among the three main categories (Control, MDD, and MDDS) were evaluated using the Kruskal–Wallis test. Statistical significance was set at p ≤ 0.05. For *post hoc* pairwise comparisons, the Mann–Whitney U test was employed, and p-values were adjusted for multiple testing using the Holm–Bonferroni correction (Chalmers et al., 2022; Morehouse et al., 2025).

## Results

### Signal decomposition and coupling framework

**FIGURE 2:**
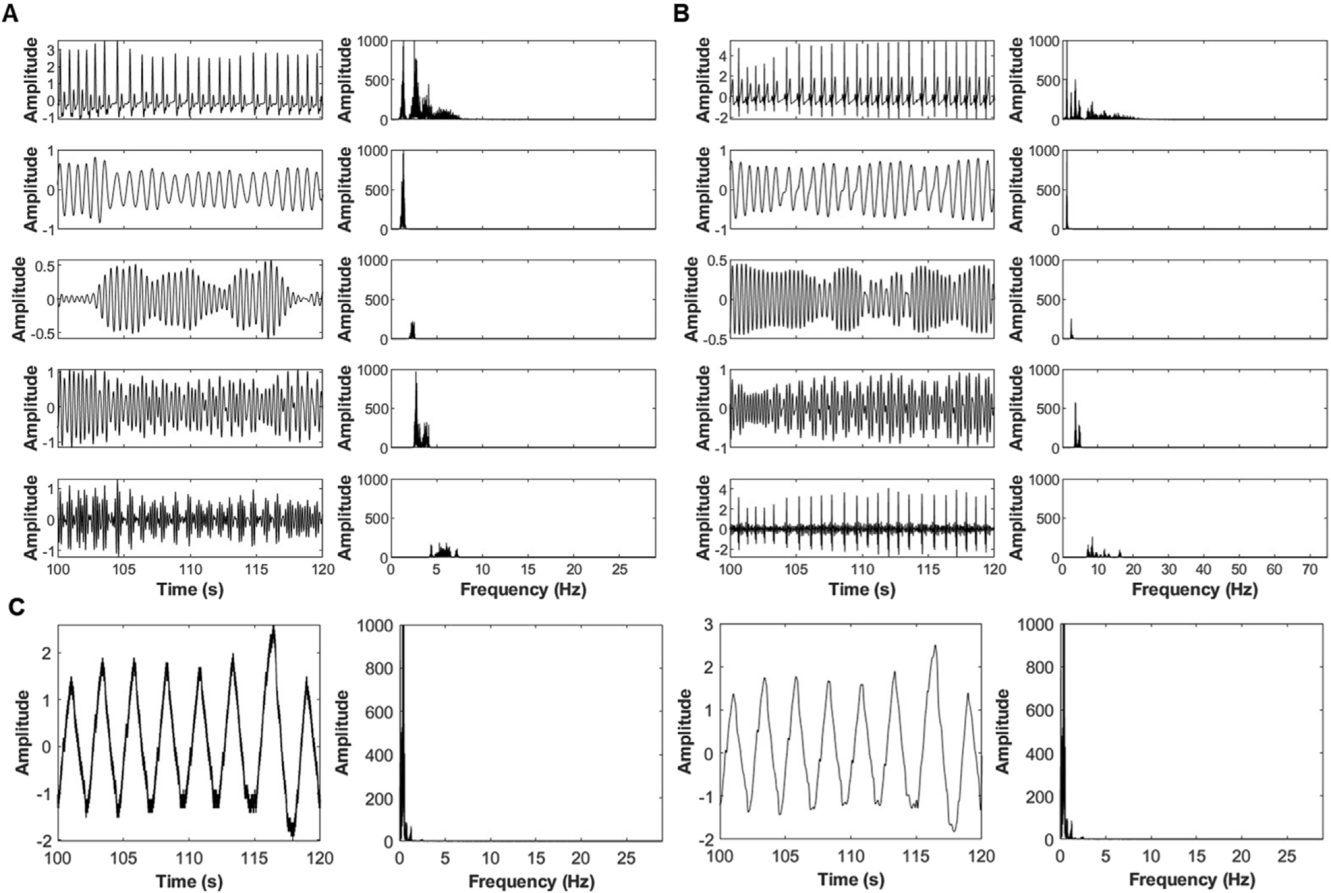
Decomposition of physiological signals using the SWD algorithm. **(A)** PPG signal, **(B)** ECG signal, **(C)** respiratory signal. Original signals (top) are divided into four oscillatory components (OCs) with low, mid-low, mid-high, and high frequencies (top to bottom). For respiration, only the low-frequency OC1 was analyzed further showing two examples of respiratory signals OC1 (left) with their corresponding frequency range (right).

Swarm Decomposition (SwD) was applied to extract four oscillatory components (OCs) from ECG, PPG, and respiratory signals, spanning low to high frequencies and characterize frequency-specific bidirectional interactions between cardiac and respiratory signals. Figure 2 illustrates this decomposition, showing the raw signals (top) and their respective OCs (bottom) for ECG, PPG, and respiration. SwD preserves physiological structure across scales, with respiration limited to its lowest frequency component (OC1) to maintain the reference driver signal. Because the decomposition is data-driven, the precise frequency ranges of OC1–OC4 vary across subjects; however, in our dataset OC1 typically contained frequencies below ∼1 Hz, OC2 in the ∼1–3 Hz range, OC3 in the ∼3–10 Hz range, and OC4 above ∼10 Hz (Figure 2 for representative spectra). These ranges should be interpreted as approximate, reflecting the relative ordering of the components rather than fixed frequency bands.

**FIGURE 3:**
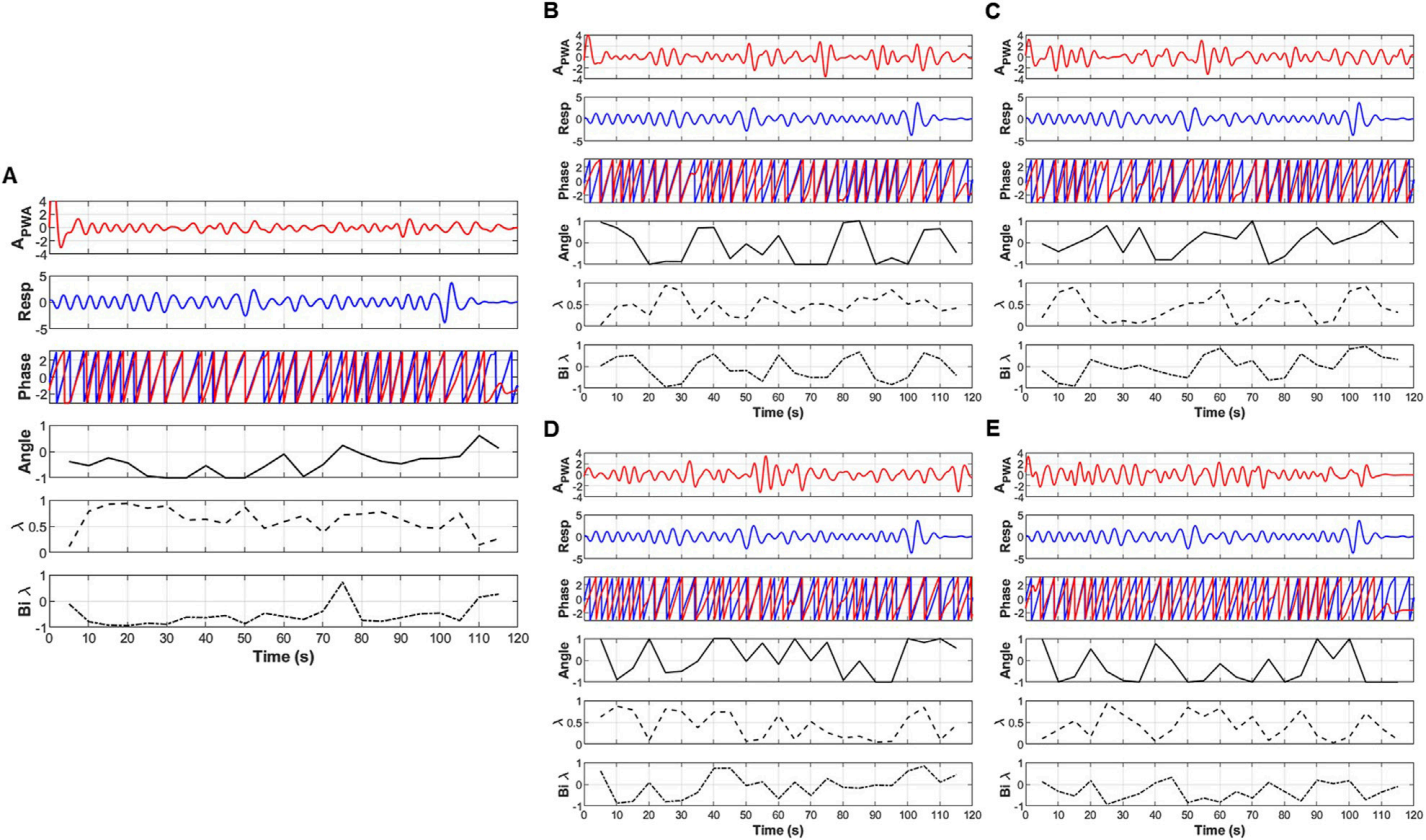
An example of the extraction of coupling information, including the angle (degree of coupling), directional coupling (λ), and bi-directional coupling (λ_bi_) between pulse wave amplitude (PWA) and respiratory signals. **(A)** original signal, **(B)** low frequency oscillatory component (OC1), **(C)** mid-low frequency OC2, **(D)** mid-high frequency OC3 and **(E)** high frequency OC4.

Coupling was computed between the respiratory signal and the three derived cardiovascular features—pulse wave amplitude (PWA), pulse transit time (PTT), and pulse rate (PR), and the three-coupling metrics: angle (phase degree), unidirectional coupling (λ), and bidirectional coupling (λ_bi_). A representative example of the coupling features across all OCs, displaying the time series, phase trajectories, and extracted coupling values (angle and λbi) is shown in Figure 3 below. The Figure indicates the distinct dynamic patterns and directional dependencies at different frequency bands, which were then quantified for statistical comparison across groups.

## Group demographical information

Subjects in the control, MDD, and MDDSI groups had average ages of 28, 34, and 32 years (Table 2). No significant difference in age was observed between the three groups. Most participants were female (66%), with male representation at 47.2%, 27.3%, and 15.8% for the control, MDD, and MDDSI groups, respectively. A significant difference was observed for gender (p = 0.014).

**TABLE 2:** Overall information of all subjects included in the study.

Variable	Control (n = 35)	MDD (n = 21)	MDDSI (n = 18)	p-value
Demographic information				
Age, yrs	28 (24.2–37.1)	34 (29.6–41.0)	32 (27.5–43.1)	0.119
Male, n	17 (47.2)	6 (27.3)	3 (15.8)	0.014
Anthropometric/physiological indices				
WC, cm	73.5 (70.0–83.2)	89.5 (78.1–101.0)	87.0 (77.8–99.0)	0.001^*o^
BMI, kg/m^2^	23.4 (21.4–26.0)	27.0 (16.5–47.1)	26.4 (23.2–31.5)	0.065
MAP, mmHg	83.3 (80.8–89.5)	86.7 (79.9–92.1)	83.3 (80.3–91.6)	0.894
Psychometric/clinical questionnaire scores				
Suicidal score	-	0 (0–3.1)	17 (13.8–22.9)	<0.001^+^
BDI	-	27.5 (23.6–39.6)	42.0 (30.2–45.8)	0.089
GAD-7	-	15.5 (11.3–17.6)	17.0 (12.0–25.1)	0.084
PHQ9	-	16.5 (14.0–20.7)	22.0 (15.5–30.2)	0.041^+^

Values are represented as either median (inter-quartile range) and for Male as n (%). Bold p-value: Significant difference (p < 0.050). *: Significant difference between control and MDD. o: Significant difference between control and MDDSI. +: Significant difference between MDD, and MDDS. MDD: Major depressive disorder. MDDSI: MDD, plus suicidal ideation. WC: Waist circumference. BMI: Body mass index. MAP: Mean arterial pressure. BDI: Beck depression inventory. GAD-7: General anxiety disorder. PHQ9: patients health questionnaire.

Significant differences were observed for waist circumference (WC) (p-value = 0.001), suicidal score (p-value <0.001), and PHQ-9 (p-value = 0.041). For WC, the significant difference was between control and MDD and between control and MDDSI, whereas the suicidal score and PHQ- 9 showed a significant difference between MDD and MDDSI.

### Unidirectional and bidirectional coupling

**FIGURE 4:**
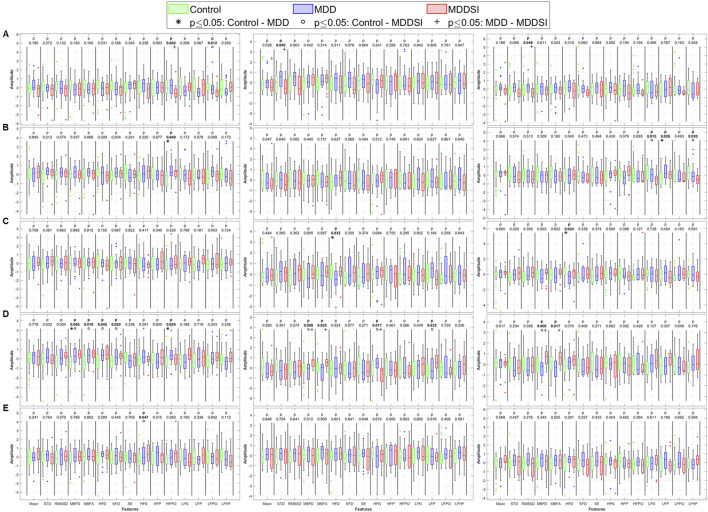
Statistical analysis of features extracted from the angle (degree of coupling) between pulse wave amplitude (PWA, left column), pulse transit time (PTT, middle column), and pulse rate (PR, right column) and respiration. **(A)** original signal, **(B)** low frequency oscillatory component (OC1), **(C)** mid-low frequency OC2, **(D)** mid-high frequency OC3, and **(E)** high frequency OC4. STD: standard deviation, RMSSD: root mean square of successive differences, MBFD: Minkowski–Bouligand box-counting fractal dimension, MBFA: Minkowski–Bouligand box-counting fractal abundance, HFD: Higuchi fractal dimension, KFD: Katz fractal dimension, SE: Shannon entropy, HFN: high frequency norm, HFP: high frequency peak, HFPO: high frequency power, LFN: low frequency norm, LFP: low frequency peak, LFPO: low frequency power, LFHF: low frequency to high frequency ratio.

Three coupling variables were analyzed: the degree or magnitude of coupling (angle), unidirectional coupling (λ), and bidirectional coupling (λ_bi_). The Kruskal–Wallis analysis of the angle for PWA, PTT, and PR uses original signals and their decomposed oscillatory components (OCs), which are shown in Figure 4. Significant differences were observed for the time-domain metrics (SDNN and RMSSD) and fractal dimension (FD) features (Minkowski–Bouligand box-counting FD and FA, and Katz FD) in the original PWA signals, between the control and MDD groups.

When analyzing higher frequency components (OC2 and OC4), significant differences were observed in LF peak for OC2 between MDD and MDDSI, and in HF power for OC4 between control and MDDSI. PTT also showed significant differences in HF norm in OC2 between control and MDD (p = 0.025). These findings suggest that frequency-specific alterations in signal complexity and autonomic tone are characteristic changes associated with different depressive states, and especially pronounced in suicidal ideation, associated with higher oscillatory levels.

**FIGURE 5:**
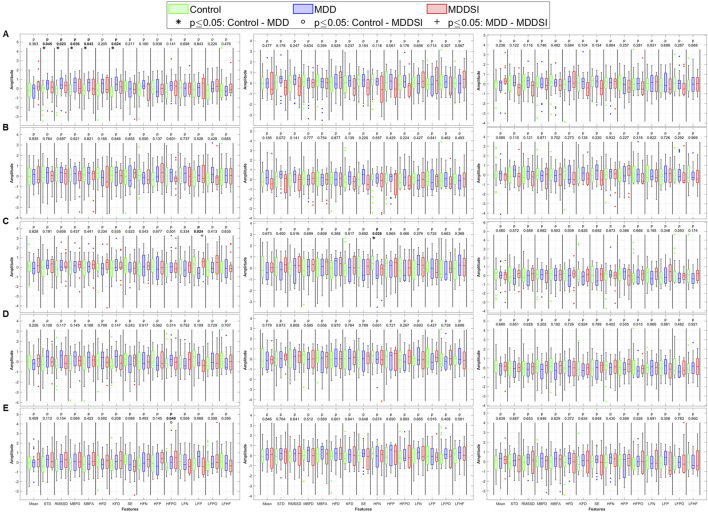
Statistical analysis of features extracted from the directional coupling (λ) between pulse wave amplitude (PWA, left column), pulse transit time (PTT, middle column), and pulse rate (PR, right column) and respiration. **(A)** original signal, **(B)** low frequency oscillatory component (OC1), **(C)** mid-low frequency OC2, **(D)** mid-high frequency OC3 and **(E)** high frequency OC4. STD: standard deviation, RMSSD: root mean square of successive differences, MBFD: Minkowski–Bouligand box-counting fractal dimension, MBFA: Minkowski–Bouligand box-counting fractal abundance, HFD: Higuchi fractal dimension, KFD: Katz fractal dimension, SE: Shannon entropy, HFN: high frequency norm, HFP: high frequency peak, HFPO: high frequency power, LFN: low frequency norm, LFP: low frequency peak, LFPO: low frequency power, LFHF: low frequency to high frequency ratio.

For directional coupling (Figure 5), significant differences emerged between MDD and MDDSI in SDNN, Shannon entropy, and frequency-domain features from the original PWA signal. As frequencies increased, more pronounced differences appeared between the control and MDD groups. The highest frequency component (OC4) revealed significant differences in PWA and PTT, particularly in low frequency norm and Higuchi FD. These alterations in OC4 imply impaired high-frequency vagal modulation and disrupted parasympathetic regulation in suicidal ideation. The entropy reduction further reflects constrained dynamical responsiveness in MDDSI subjects.

### Bidirectional coupling

**FIGURE 6:**
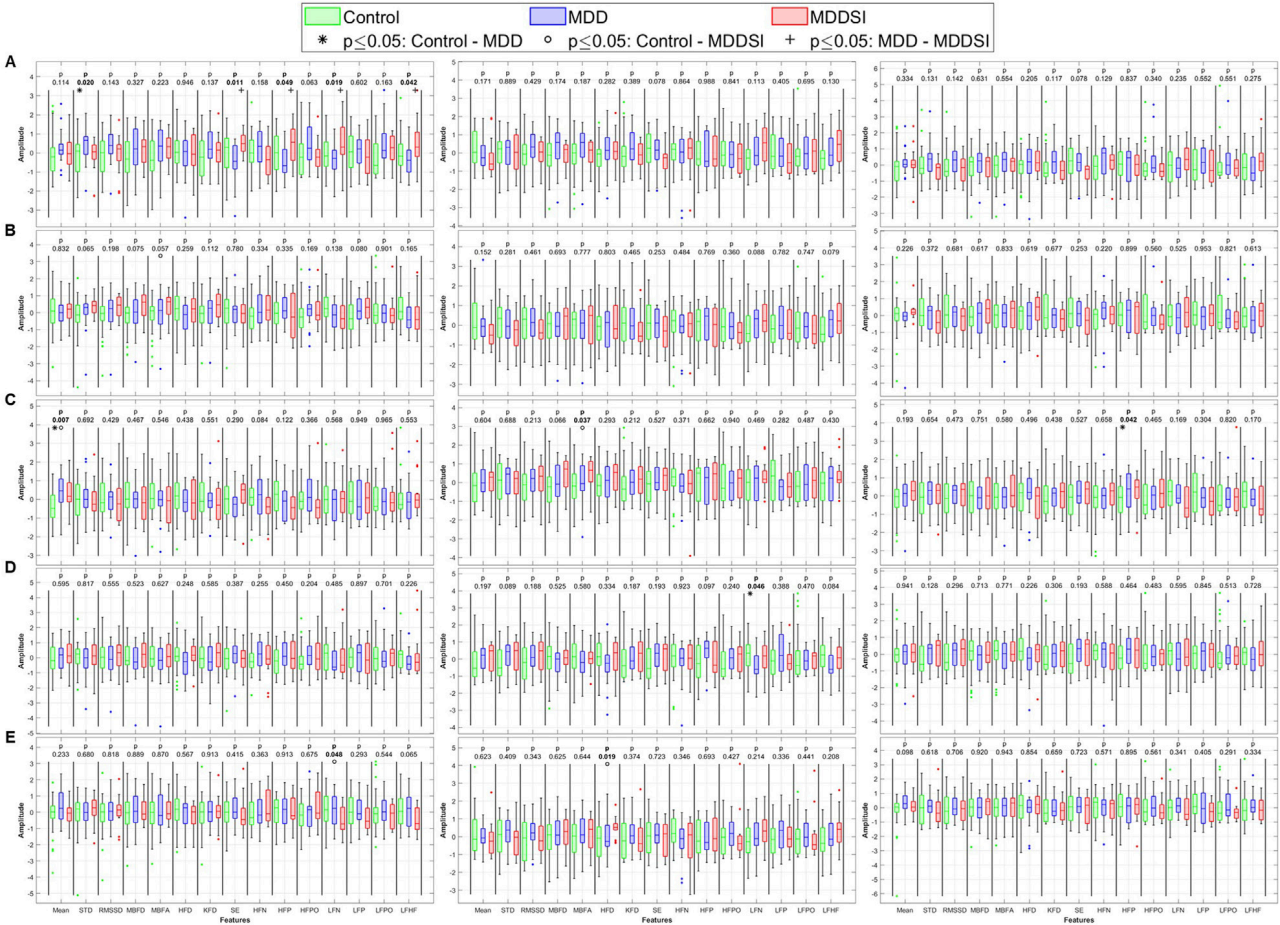
Statistical analysis of features extracted from the bi-directional coupling (λ_bi_) between pulse wave amplitude (PWA, left column), pulse transit time (PTT, middle column), and pulse rate (PR, right column) and respiration. **(A)** original signal, **(B)** low frequency oscillatory component (OC1), **(C)** mid-low frequency OC2, **(D)** mid-high frequency OC3 and **(E)** high frequency OC4. STD: standard deviation, RMSSD: root mean square of successive differences, MBFD: Minkowski–Bouligand box-counting fractal dimension, MBFA: Minkowski–Bouligand box-counting fractal abundance, HFD: Higuchi fractal dimension, KFD: Katz fractal dimension, SE: Shannon entropy, HFN: high frequency norm, HFP: high frequency peak, HFPO: high frequency power, LFN: low frequency norm, LFP: low frequency peak, LFPO: low frequency power, LFHF: low frequency to high frequency ratio.

Bidirectional coupling analysis (Figure 6) revealed more significant differences for PR than for angle or unidirectional coupling. These differences were more widely distributed across all OCs and spanned multiple physiological features. Specifically, PR in OC3 showed strong discriminatory power between the groups in both fractal and frequency domains. Shannon entropy and LF norm were significantly altered in MDDSI, highlighting the potential of mid-high frequency bidirectional coupling as a sensitive marker for suicidal ideation. Additionally, PTT in OC3 distinguished MDD from MDDSI via significant changes in Higuchi and Katz fractal dimensions, indicating increased signal irregularity and reduced complexity in more severe depressive states. These findings underscore the diagnostic value of OC3 in network physiology.

### Distribution of significant differences

**FIGURE 7:**
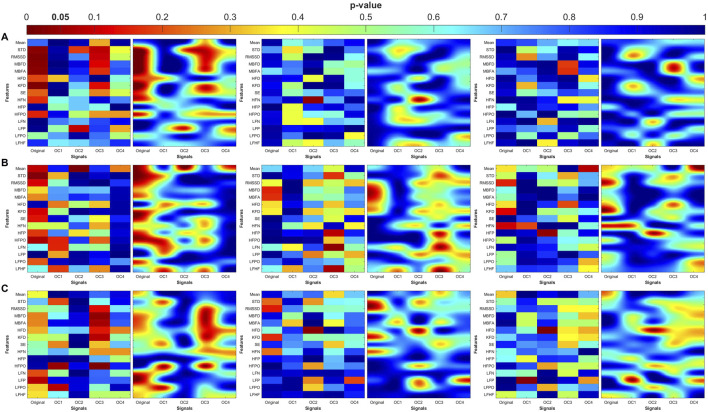
Heatmaps of the statistical p-value obtained using the time-domain, fractal dimension (FD), and frequency-domain features between the three categories (control, MDD, and MDDSI). **(A)** Pulse wave amplitude (PWA), **(B)** pulse transit time (PTT), and **(C)** pulse rate (PR). The left column shows the angle (degree of coupling), the middle column is the directional coupling (λ), and the right column is the bi-directional coupling (λ_bi_).

**FIGURE 8:**
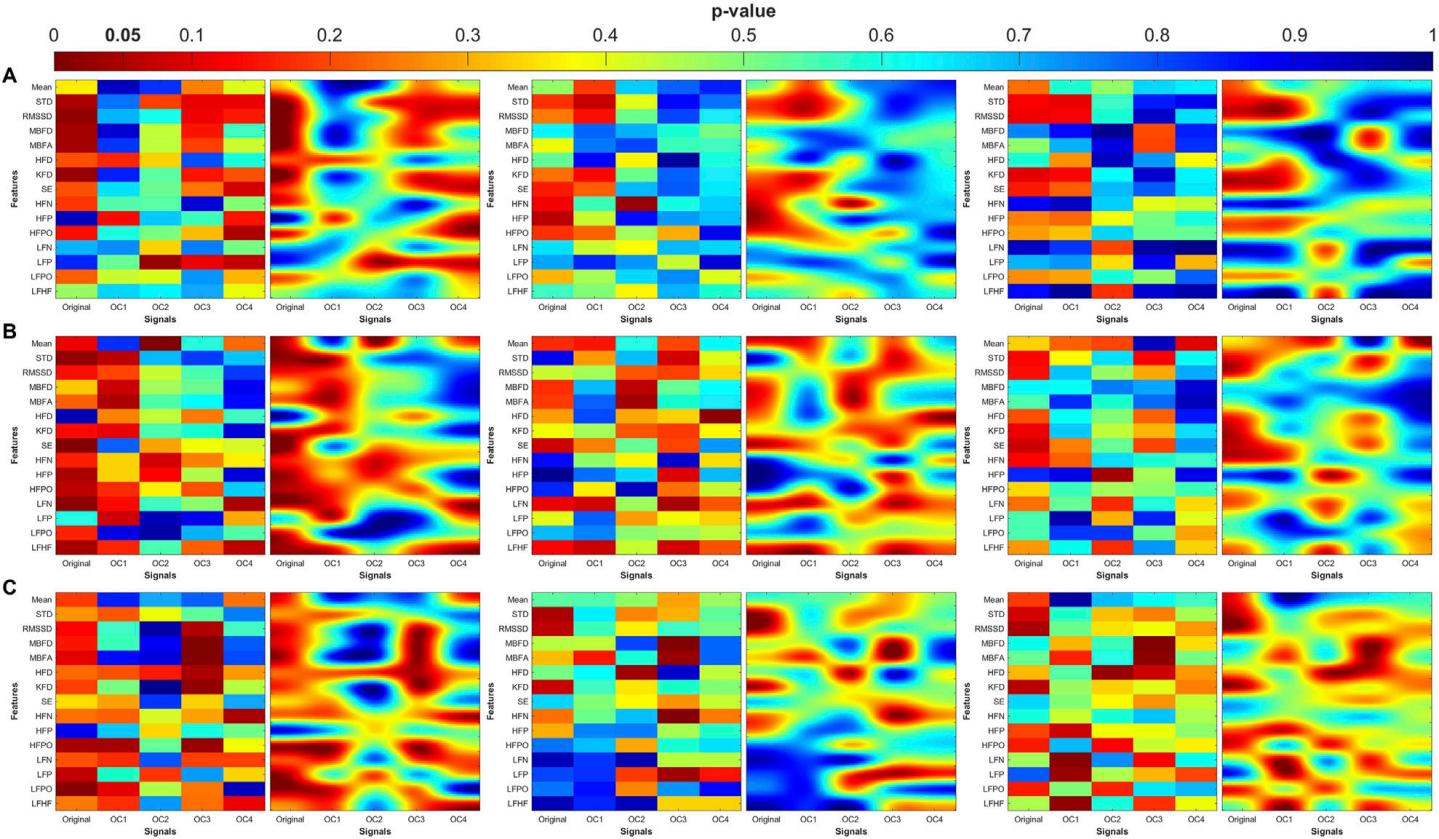
Heat maps of the statistical p-value were obtained using the time-domain, fractal dimension (FD), and frequency-domain features of the control and MDD. **(A)** Pulse wave amplitude (PWA), **(B)** pulse transit time (PTT) and **(C)** pulse rate (PR). The left column shows the angle (degree of coupling), the middle column is the directional coupling (λ), and the right column is the bi-directional coupling (λ_bi_).

**FIGURE 9:**
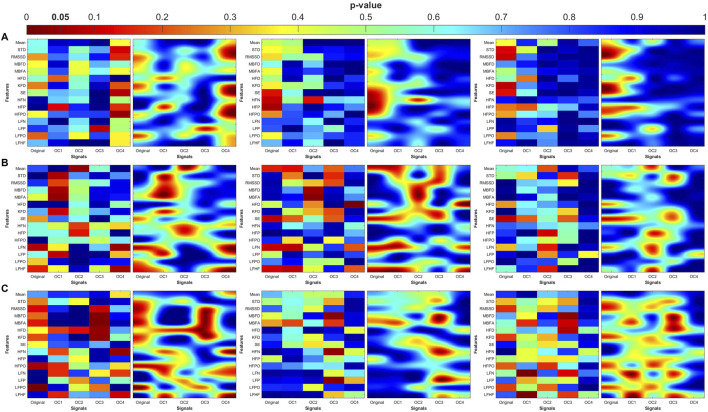
Heat maps of the statistical p-value were obtained using the time-domain, fractal dimension (FD), and frequency-domain features of the control and MDDSI. **(A)** Pulse wave amplitude (PWA), **(B)** pulse transit time (PTT) and **(C)** pulse rate (PR). The left column shows the angle (degree of coupling), the middle column is the directional coupling (λ), and the right column is the bi-directional coupling (λ_bi_).

**FIGURE 10:**
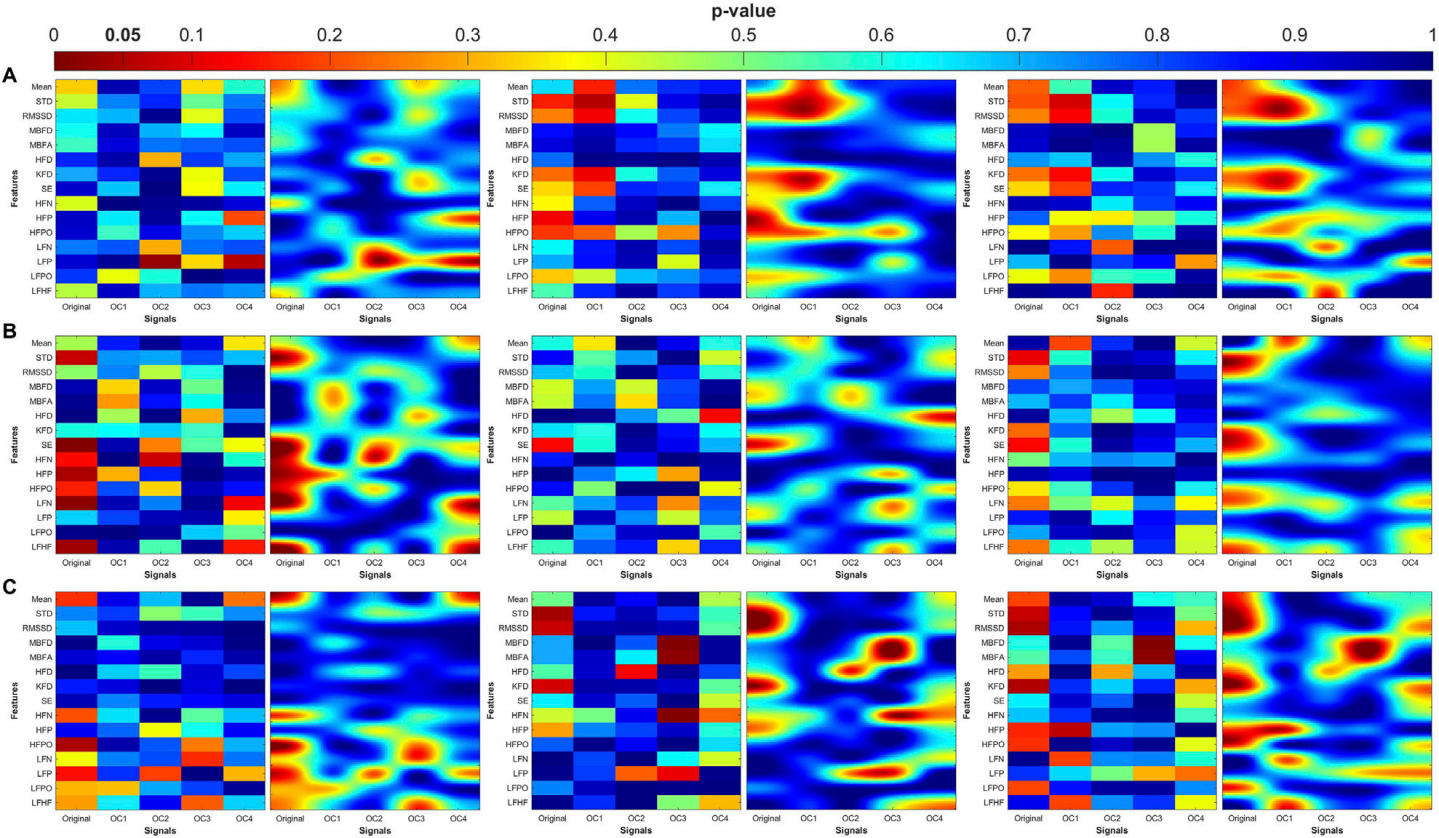
Heat maps of the statistical p-value were obtained using the time-domain, fractal dimension (FD), and frequency-domain features of the MDD and MDDSI. **(A)** Pulse wave amplitude (PWA), **(B)** pulse transit time (PTT) and **(C)** pulse rate (PR). The left column shows the angle (degree of coupling), the middle column is the directional coupling (λ), and the right column is the bi-directional coupling (λ_bi_).

The heatmaps in Figures 7–10 illustrate the distribution of significant p-values across all features and OCs. Figure 7 highlights that the most consistent differences were observed across all coupling modes in OC3 and OC4 for both PR and PTT, confirming their importance in differentiating MDD and MDDSI from controls. Figure 7 further shows that MDDSI is marked by widespread disruptions in bi-directional coupling (λ_bi_), particularly in high-frequency features and entropy measures of PR and PTT, not evident in MDD alone.

The heat maps revealed that significant group differences were consistently localized in the mid-high (OC3) and high-frequency (OC4) components, particularly for pulse rate (PR) and pulse transit time (PTT) under bi-directional coupling (λ_bi_). Features such as Shannon entropy, Higuchi fractal dimension, and high-frequency power showed strong discriminatory capacity between MDD and MDDSI. These patterns highlight frequency-specific disruptions in physiological complexity and coupling directionality associated with increasing psychiatric severity.

## Discussion

The present study provides novel insights into physiological network changes associated with major depressive disorder (MDD) and suicidal ideation (MDDSI), with a focus on frequency-specific bidirectional cardio-respiratory coupling and providing a framework for psychiatric network physiology. Our findings were consistent with ongoing evidence in literature that there is robust cardiorespiratory coupling in adults, underscoring the relevance of physiological synchronization, even in non-pathological populations (Sobiech et al., 2017). The integration of Swarm Decomposition (SwD) with phase-based coupling metrics, fractal geometry, and entropy analysis enabled a detailed, scale-sensitive characterization of autonomic dysfunction of depressive phenotypes. Moreover, although transfer entropy is an established information-theoretic approach for quantifying directed interactions, it was not directly employed as a feature in this study. Instead, we extracted measures characterizing fractal properties, entropy, and frequency-domain parameters, in addition to basic statistical descriptors. This choice reflects our focus on fractal and oscillatory properties of phase coupling, while acknowledging that transfer entropy represents a complementary approach for future analyses. In addition to these nonlinear descriptors, we also calculated linear parameters (mean and standard deviation) for each oscillatory component (OC1–OC4). This allowed us to evaluate both linear and nonlinear properties of the coupling dynamics when comparing groups.

Previous research has reported reduced heart rate variability (HRV) and impaired respiratory sinus arrhythmia (RSA) as hallmarks of MDD (Brunoni et al., 2013; Wainsztein et al., 2020). However, unlike traditional HRV or RSA indices, which are limited to global measures and unidirectional influences, this study demonstrates that mid- and high-frequency oscillatory components (OC3 and OC4) in cardio-respiratory signals—particularly pulse rate (PR) and pulse transit time (PTT) that carry discriminative value in differentiating MDD from MDDSI. Specifically, the bidirectional coupling strength (λ_bi_) of PR in OC3 yielded significant group differences, while the reduced Higuchi fractal dimension associated with PTT in MDDSI suggests a collapse in physiological complexity. These results extend previous findings of diminished fractal scaling in subthreshold or overt depressive states (Mandarano et al., 2022; Valenza et al., 2015; Bartsch et al., 2014). The directional entropy differences observed between MDD and MDDSI agree with prior entropy-based studies on affective states, where reduced entropy reflected diminished variability between both groups and increased physiological rigidity (Sheridan et al., 2021; Čukić et al., 2023). This is particularly relevant when considering the role of entropy in predicting suicidal ideation linked to altered photoplethysmographic entropy dynamics (Khandoker et al., 2017a; Khandoker et al., 2017b). The introduction of both oscillatory components and spectral indices provided a multifaceted view of coupling dynamics. Although this finer stratification makes direct physiological interpretation more complex, it also offers exploratory value by uncovering patterns that may support state discrimination. Such an approach complements established methodologies and may open avenues for refining feature selection in future studies. Moreover, λ(tk) and Biλ(tk) are nonlinear indices of cardiorespiratory coupling75. The additional features extracted, e.g., fractal dimensions, entropy, and spectral indices, were therefore considered as descriptors of the variability and structural properties of the λ(tk) time series. This approach was adopted to maximize discriminatory potential between groups and provide a summary of these time series in the form of extracted features.

While earlier approaches like Granger causality and transfer entropy have been instrumental in identifying directionality in physiological networks, they often suffer from high computational demands and limited applicability to nonstationary data (Abdul Razak and Jensen, 2014). The current study addresses these issues by extending the Niizeki-Saitoh phase coherency algorithm into a bidirectional coupling framework that is both computationally efficient and physiologically interpretable (Niizeki and Saitoh, 2012). The suggested λ_bi_, further improves inconclusive or weak cardiorespiratory interactions using traditional long-range correlation techniques (Valenza et al., 2018a; Faes et al., 2013). Furthermore, the use of SwD provided an advantage over conventional decomposition methods such as empirical mode decomposition or variational mode decomposition, which may introduce mode mixing or fail to adapt to signal-specific frequency characteristics (Saleem et al., 2022). SwD preserved signal integrity and allowed for more accurate identification of functionally relevant oscillatory components.

The adaptive decomposition used by SwD was essential in isolating frequency bands that correspond to autonomic nervous system components, and integrative cortical-subcortical dynamics, which reflect hierarchical models of autonomic regulation in psychiatric illness (Lahey et al., 2021). The current results suggest that MDDSI is characterized not only by a further degradation of vagal regulation and signal complexity compared to MDD, but also by a specific pattern of disrupted bidirectional interaction across oscillatory bands. This reinforces the hypothesis that suicidality represents a physiologically distinct phenotype within depressive disorders, with implications for diagnostic stratification and targeted intervention.

## Limitations

This study provides novel insights into frequency-specific bidirectional cardio-respiratory coupling in depression and suicidal ideation. However, several limitations need to be mentioned. However, there are limitations. The sample size was relatively small, particularly in the MDDSI group, potentially limiting statistical power and generalizability. Additionally, the use of only resting-state data and the available two-minute signal segments influence the detection of longer-range dynamics. Despite these constraints, the findings indicate the clinical potential of Swarm Decomposition and bidirectional coupling metrics, specifically λ_bi_ in OC3 and OC4, as physiological biomarkers for stratifying depressive phenotypes for clinical decision making and treatment options. Use of multimodal and bidirectional coupling has shown their importance in differentiating MDD from MDDSI through entropy and fractal features and incorporating multiscale network physiology into digital psychiatry. Moreover, we did not examine spectral measures of GC, which extend GC into the frequency domain and may provide additional insights into oscillatory interactions between physiological systems (Faes et al., 2022; Chicharro, 2011; Bilgin et al., 2009). Future work is aimed at replicating these results in larger, longitudinal cohorts, integrating multimodal data (e.g., EEG, behavioral indices), and exploring how these metrics respond to therapeutic interventions.

## Conclusion

In this study, we examined coupling dynamics across multiple oscillatory components to differentiate between MDD subtypes and healthy controls. This study shows that frequency-specific disruptions in bidirectional cardiorespiratory coupling, together with reductions in signal complexity and entropy, distinguish MDDSI from other groups. These findings suggest impaired autonomic adaptability and emotional regulation in this high-risk depressive state. By combining phase-based coupling metrics with SwD analysis, our work highlights promising physiological markers that may support early identification and stratification of depressive subtypes in digital psychiatry. Future research should aim to validate these markers in larger cohorts and refine their clinical applicability.

## Data Availability

The raw data supporting the conclusions of this article will be made available by the authors, without undue reservation.
